# Foot-and-mouth disease virus induces lysosomal degradation of NME1 to impair p53-regulated interferon-inducible antiviral genes expression

**DOI:** 10.1038/s41419-018-0940-z

**Published:** 2018-08-29

**Authors:** Huan-Huan Feng, Zi-Xiang Zhu, Wei-Jun Cao, Fan Yang, Xiang-Le Zhang, Xiao-Li Du, Ke-Shan Zhang, Xiang-Tao Liu, Hai-Xue Zheng

**Affiliations:** State Key Laboratory of Veterinary Etiological Biology, National Foot and Mouth Diseases Reference Laboratory, Key Laboratory of Animal Virology of Ministry of Agriculture, Lanzhou Veterinary Research Institute, Chinese Academy of Agricultural Sciences, Lanzhou, Gansu P. R. China

## Abstract

Nucleoside diphosphate kinase 1 (NME1) is well-known as a tumor suppressor that regulates p53 function to prevent cancer metastasis and progression. However, the role of NME1 in virus-infected cells remains unknown. Here, we showed that NME1 suppresses viral replication in foot-and-mouth disease virus (FMDV)-infected cells. NME1-enhanced p53-mediated transcriptional activity and induction of interferon-inducible antiviral genes expression. FMDV infection decreased NME1 protein expression. The 2B and VP4 proteins were identified as the viral factors that induced reduction of NME1. FMDV 2B protein has a suppressive effect on host protein expression. We measured, for the first time, VP4-induced lysosomal degradation of host protein; VP4-induced degradation of NME1 through the macroautophagy pathway, and impaired p53-mediated signaling. p53 plays significant roles in antiviral innate immunity by inducing several interferon-inducible antiviral genes expression, such as, *ISG20*, *IRF9*, *RIG-I,* and *ISG15*. VP4 promoted interaction of p53 with murine double minute 2 (MDM2) through downregulation of NME1 resulting in destabilization of p53. Therefore, 5-flurouracil-induced upregulation of *ISG20*, *IRF9*, *RIG-I*, and *ISG15* were suppressed by VP4. VP4-induced reduction of NME1 was not related to the well-characterized blocking effect of FMDV on cellular translation, and no direct interaction was detected between NME1 and VP4. The 15–30 and 75–85 regions of VP4 were determined to be crucial for VP4-induced reduction of NME1. Deletion of these VP4 regions also inhibited the suppressive effect of VP4 on NME1-enhanced p53 signaling. In conclusion, these data suggest an antiviral role of NME1 by regulation of p53-mediated antiviral innate immunity in virus-infected cells, and reveal an antagonistic mechanism of FMDV that is mediated by VP4 to block host innate immune antiviral response.

## Introduction

Foot-and-mouth disease (FMD) is a highly contagious disease that mainly affects pigs, cows, sheep, goats, deer, and other cloven-hoofed animals. The causative agent of FMD is the foot-and-mouth disease virus (FMDV), which belongs to the genus *Aphthovirus* of family *Picornaviridae*. The viral genome contains a positive single-strand RNA chain ~8.5 kb in length, encoding four structural proteins, eight non-structural proteins and some cleavage intermediates. The viral proteins perform a variety of functions to enable the spread and replication of FMDV and counteract host antiviral responses through many different mechanisms.

Nucleoside diphosphate kinase 1 (NME1, also known as NM23-H1) has a metastasis-suppressive function in several malignancies^1–3^. In recent years, NME1 has been ascribed numerous functions that are inconsistent with its previous functions. It is suggested that NME1 has an effect on the cell-adhesion-related signaling pathway and plays critical roles in cellular proliferation, signal transduction, growth control, embryonic development, differentiation, and oncogenesis^4,5^. However, the exact mechanism by which NME1 suppresses metastasis is unknown. It is proposed that NME1 interacts with the macrophage migration inhibitory factor (MIF), which is a pluripotent cytokine involved in host immune and inflammatory responses and tumorigenesis^6^. This interaction between NME1 and MIF may result in a negative regulatory effect of NME1 on tumor metastasis.

Tumor virus infection can result in oncogenic tumor initiation. Epstein-Barr virus (EBV) is one of the most common viruses associated with particular forms of cancer. EBV-derived nuclear antigen 1 and nuclear protein EBNA-3C both interact with NME1 and reverse the metastasis-suppressive activity of NME1, indicating that these EBV proteins target and antagonize the functions of NME1 to promote cancer metastasis^7,8^. Recent data suggest an essential role for NME1 in the suppression of tumor-virus-induced cell migration and cancer progression^9^. The interactions between NME1 and viral oncoproteins are apparently associated with this suppressive role.

The tumor suppressor protein, p53, is widely known as ‘the guardian of the genome’ due to its ability to prevent the emergence of transformed cells^10,11^. In addition, p53 is involved in the host antiviral defense in many viral infections^12,13^. As a transcriptional factor, p53 plays significant roles in antiviral innate immunity by promoting the interferon (IFN) pathway activation and by inducing several antiviral proteins expression^14,15^. NME1, acts as an anti-metastatic factor, and interacts with p53 in the suppression of cancer progression^16,17^. However, the state and function of NME1 in virus-infected cells and whether NME1 has a potential suppressive role against viruses in infected cells, as well as whether it is involved in the host antiviral response regulated by p53 remain unclear.

In this study, we determined that NME1 enhanced the p53-mediated antiviral response and significantly suppressed FMDV replication. FMDV VP4 and 2B are suggested to inhibit NME1-mediated antiviral activity; therefore, we determined the antiviral role and mechanism of NME1, as well as a novel antagonistic mechanism for FMDV.

## Results

### FMDV infection suppresses NME1 protein expression

**Fig. 1:**
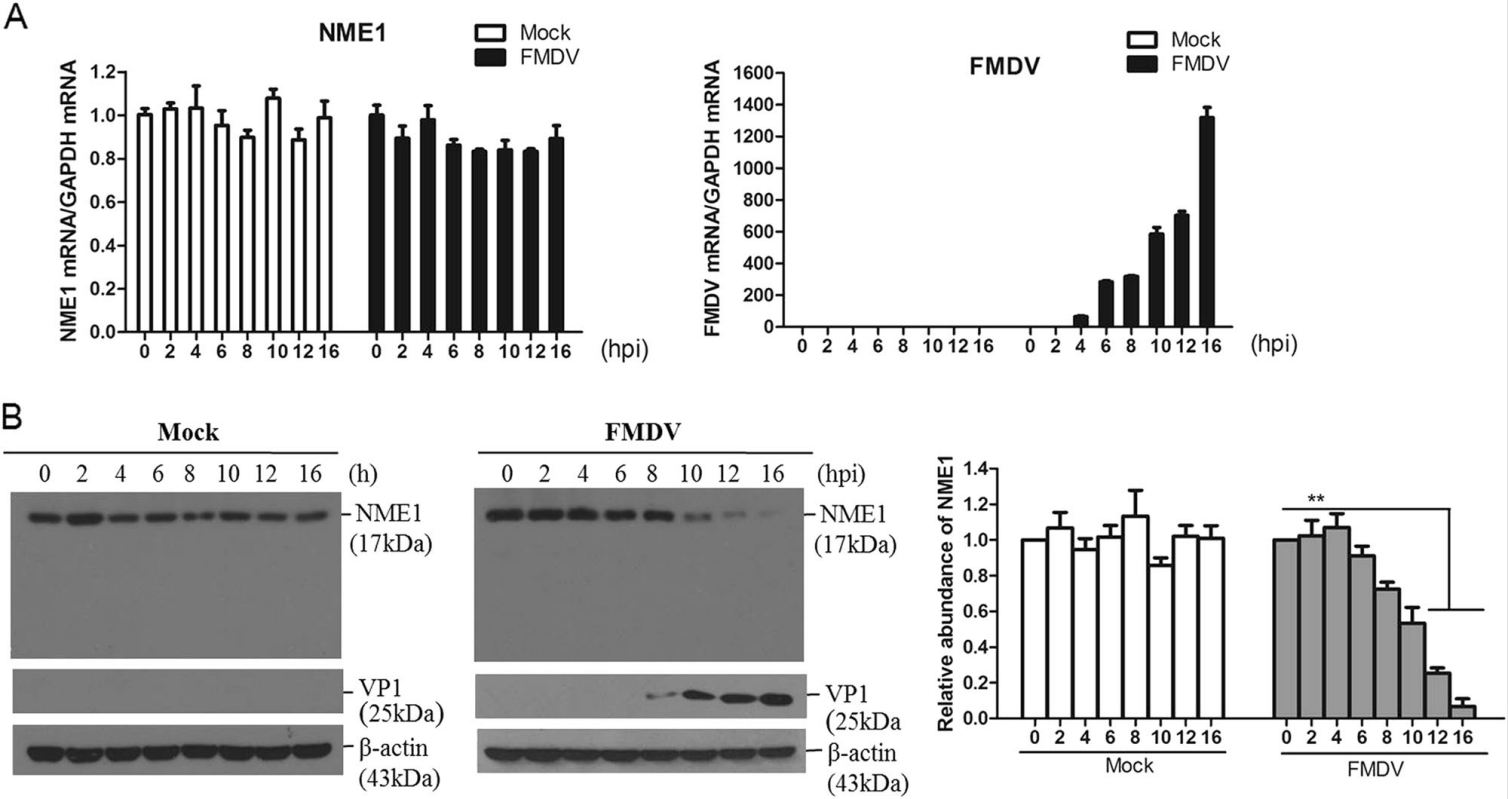
FMDV infection decreases NME1 protein expression. PK-15 cells cultured in 35-mm dishes were mock-infected or infected with 1 MOI FMDV. The cells were collected at 0, 2, 4, 6, 8, 10, 12, and 16 hpi. **a** The transcripts of NME1 and viral RNA were analyzed by qPCR. **b** Expression of NME1 and viral proteins were detected by Western blotting. The relative abundance of VP1 protein was used as an indicator of viral replication. Relative fold-change in abundance of NME1 in mock-infected or FMDV-infected cells was determined by densitometric analysis and normalized to β-actin. All the above experiments were repeated at least three times

To investigate the state of NME1 in FMDV-infected cells, PK-15 cells were mock-infected or infected with FMDV at different times; and NME1 transcript and protein levels were examined by quantitative polymerase chain reaction (qPCR) and Western blotting, respectively. FMDV infection had no effect on NME1 transcripts at 16 h post-infection (hpi), and no significant changes in NME1 mRNA levels were observed in mock-infected cells (Fig. 1a). However, in FMDV-infected cells, NME1 protein levels decreased after 8 hpi, which became more marked as the infection progressed (8–16 h) and no cleaved bands were observed **(**Fig. 1b). No marked changes in NME1 protein levels were detected in mock-infected cells (Fig. 1b). Similar results were observed with O/BY/CHA/2010 and A/HuBWH/CHA/2009 FMDV strains; therefore, we showed only the results obtained from O/BY/CHA/2010.

### NME1 plays an important role in suppression of FMDV replication

**Fig. 2:**
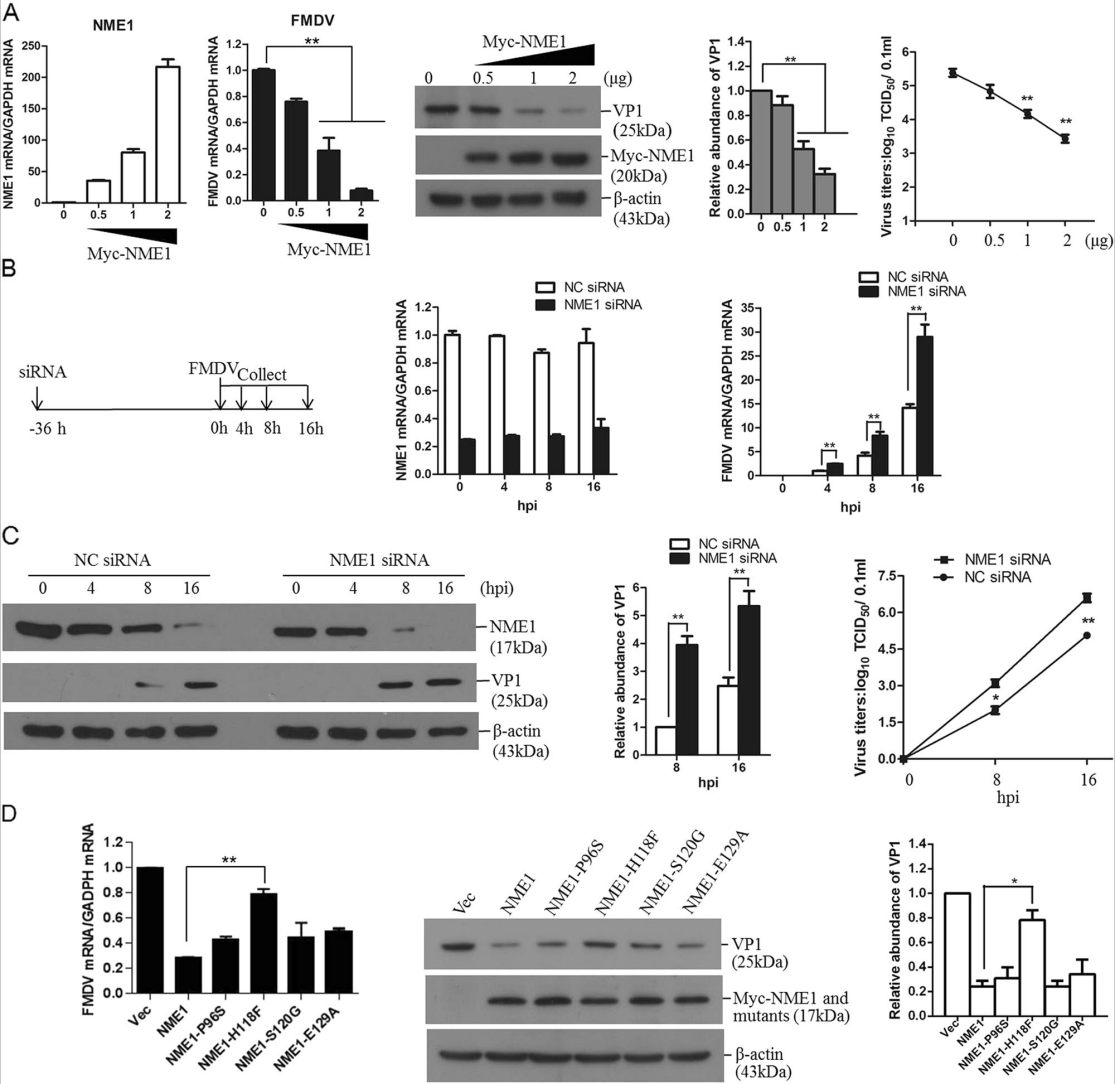
NME1 possesses antiviral function against FMDV. **a** PK-15 cells were transfected with 0, 0.5, 1, or 2 μg of Myc-tagged NME1-expressing plasmids. Empty vector was used in the transfection process to ensure that the cells received the same amount of total DNA plasmids. Transfected cells were infected with equal amounts of FMDV at 24 hpt. Infected-cells were collected at 12 hpi. NME1 mRNA and viral RNA were analyzed by qPCR. Myc-NME1 and FMDV VP1 proteins were detected by Western blotting. Relative fold-change in abundance of VP1 protein was determined by densitometric analysis. Viral titers were measured by TCID_50_ assay. **b**, **c** PK-15 cells were transfected with 150 nM of NC siRNA or NME1 siRNA for 36 h. The transfected cells were infected with FMDV and collected at 0, 4, 8, or 16 hpi. **b** Schematics of the process used in the RNAi assay and confirmation of the efficiency of NC siRNA and NME1 siRNA in silencing NME1 expression by qPCR. Viral RNA in the NC siRNA and NME1 siRNA cells was compared by qPCR analysis. **c** Viral protein and NME1 protein in NC siRNA and NME1 siRNA cells were detected by Western blotting (left panel). Relative levels of VP1 protein are indicated in folds after normalization with β-actin (middle panel). The viral titers were measured by TCID_50_ assay (right panel). **d** PK-15 cells were transfected with 2 μg of empty vector, Myc-NME1 or NME1 mutant expressing plasmids (P96S, H118F, S120G, and E129A) for 24 h. The cells were then infected with equal amounts of FMDV for 12 h. The viral RNA were analyzed by qPCR. Myc-NME1, the NME1 mutants, and FMDV VP1 proteins were detected by Western blotting. Relative fold-change in abundance of VP1 protein was determined by densitometric analysis. All the above experiments were repeated three times

FMDV infection downregulated NME1 protein levels, which implied a role of NME1 during FMDV infection. To explore the potential effect of NME1 on FMDV replication, PK-15 cells were transfected with different amounts of plasmids expressing Myc-NME1, and empty vector plasmids were used in the transfection process to ensure the cells received the same amounts of total plasmids. At 24 hpt, transfected cells were infected with equal amounts of FMDV for 12 h, and the viral RNA levels, protein abundance, and yields were detected and compared. Expression of Myc-NME1 was confirmed by qPCR and Western blotting, respectively. Overexpression of NME1 significantly suppressed FMDV replication in a dose-dependent manner (Fig. 2a). The replicative status of FMDV in NME1 knockdown cells was also examined. Knockdown of NME1 markedly promoted FMDV replication (Fig. 2b). The viral proteins and yields were further evaluated and analyzed in NME1 knockdown cells, which also indicated that downregulation of NME1 significantly enhanced FMDV replication (Fig. 2c). These results suggest an important role for NME1 in suppression of FMDV replication in host cells.

A series of NME1 mutants were further generated as described previously to investigate if the catalytic activity of the NME1 kinase is important for its anti-viral activity during FMDV infection^18,19^, and the overexpression assays were performed. Overexpression of NME1, NME1-P96S, NME1-S120G, and NEM1-E129A all significantly suppressed FMDV replication. However, overexpression of NME1-H118F only slightly suppressed FMDV replication (Fig. 2d). This suggests that the catalytic histidine at 118 site is involved in the antiviral role of NME1.

### NME1 enhances p53-mediated transcriptional activity and induction of IFN-inducible antiviral gene expression

**Fig. 3:**
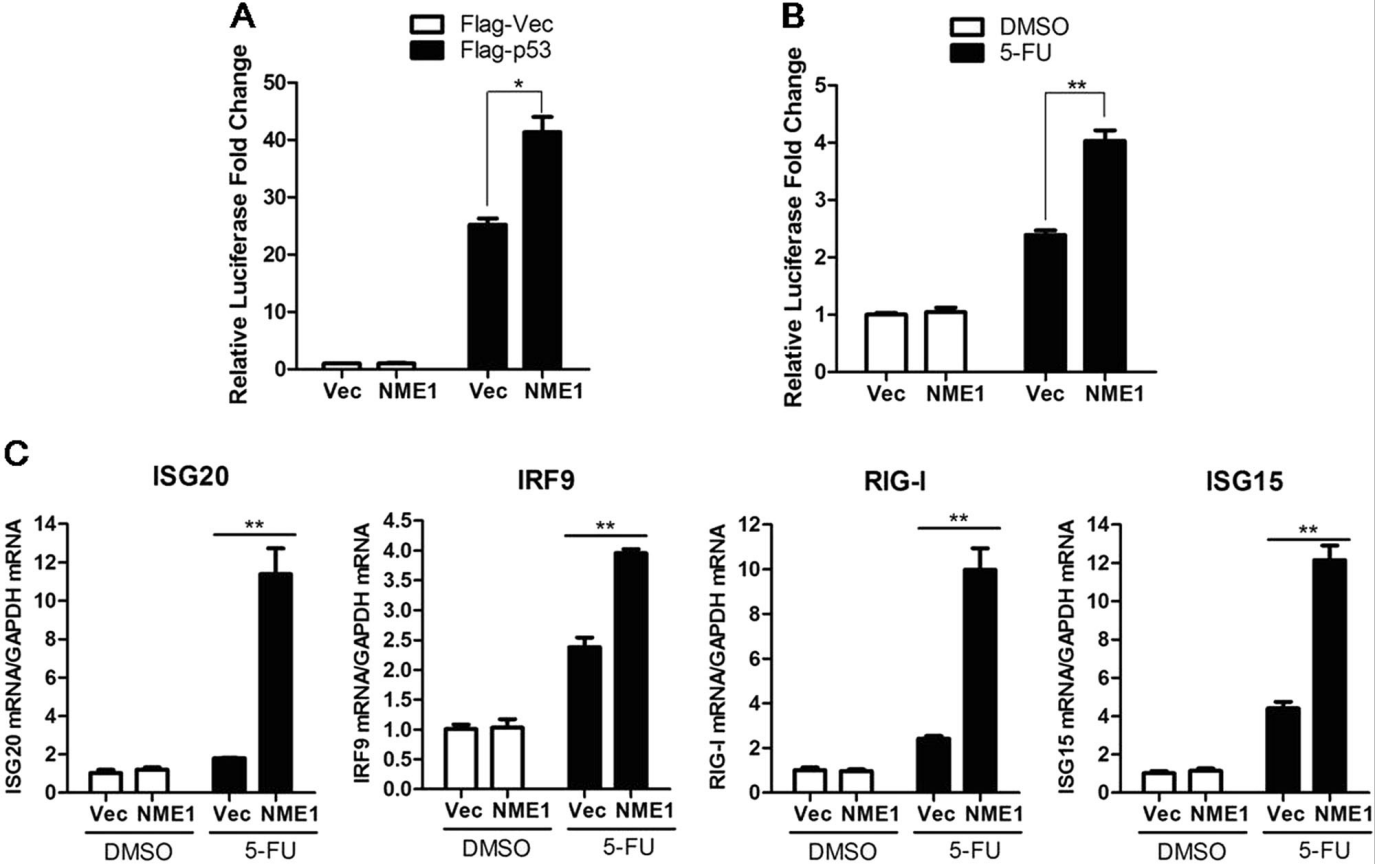
NME1 enhances p53 transcriptional function. **a** HEK-293T cells cultured in 24-well plates were co-transfected with 0.2 μg Myc-vector or 0.2 μg Myc-NME1 and 0.1 μg Flag-vector or 0.1 μg Flag-p53 plasmids, along with 0.1 μg p53 luciferase reporter plasmid. pRL-TK *Renilla* luciferase reporter plasmid (0.01 μg) was used in the reporter assay to normalize transfection efficiency. Cells were collected at 24 hpt and luciferase activities were measured using the dual-specific luciferase assay kit. **b** HEK-293T cells cultured in 24-well plates were co-transfected with 0.2 μg Myc-vector or 0.2 μg Myc-NME1 along with 0.1 μg p53 luciferase reporter and 0.01 μg pRL-TK plasmids. The transfected cells were incubated with DMSO or 5-FU (20 μg/mL) at 6 hpt, and luciferase activities were measured at 24 h after incubation. **c** HEK-293T cells cultured in six-well plates were co-transfected with 2 μg Myc-vector or 2 μg Myc-NME1, and the transfected cells were treated with DMSO or 5-FU (20 μg/mL) at 6 hpt for another 24 h. The mRNA expression levels of p53 target genes ISG20, IRF9, RIG-I, and ISG15 were analyzed by qPCR. All the experiments were repeated three times

NME1 interacts with p53 and regulates p53-mediated functions^16^. The effect of NME1 on p53-mediated transcriptional activity and induction of IFN-inducible antiviral genes was determined to evaluate the relationship between NME1 and p53 in the antiviral response. HEK-293T cells were co-transfected with Myc-vector or Myc-NME1, and Flag-vector or Flag-p53-expressing plasmids in the presence of the p53-Luc reporter and pRL-TK plasmids. Transfectants were harvested at 24 hpt and subjected to luciferase assays. Flag-p53-triggered p53-Luc promoter activity was markedly enhanced by NME1 (Fig. 3a). 5-Fluorouracil (5-FU) has been widely used to induce cellular p53 stabilization and activation of p53-mediated signaling^20^. The effect of NME1 on 5-FU-induced p53-Luc promoter activation was also evaluated. HEK-293T cells were incubated with 5-FU or a solvent control (dimethyl sulfoxide; DMSO) in the presence of the p53-Luc reporter and pRL-TK plasmids. p53-Luc promoter activity was examined at 24 h after incubation. 5-FU-triggered p53-Luc promoter activity was enhanced by NME1 (Fig. 3b). IFN-inducible genes, including *ISG20*, *IRF9*, *RIG-I*, and *ISG15* are p53 direct transcriptional targets that directly participate in host innate antiviral response. 5-FU-triggered upregulation of these genes was also determined, with results indicating that NME1 overexpression significantly enhanced expression of these genes (Fig. 3c). These results suggest that NME1 enhances p53-mediated function in the host antiviral response.

### FMDV VP4 and 2B proteins suppress NME1 protein expression

**Fig. 4:**
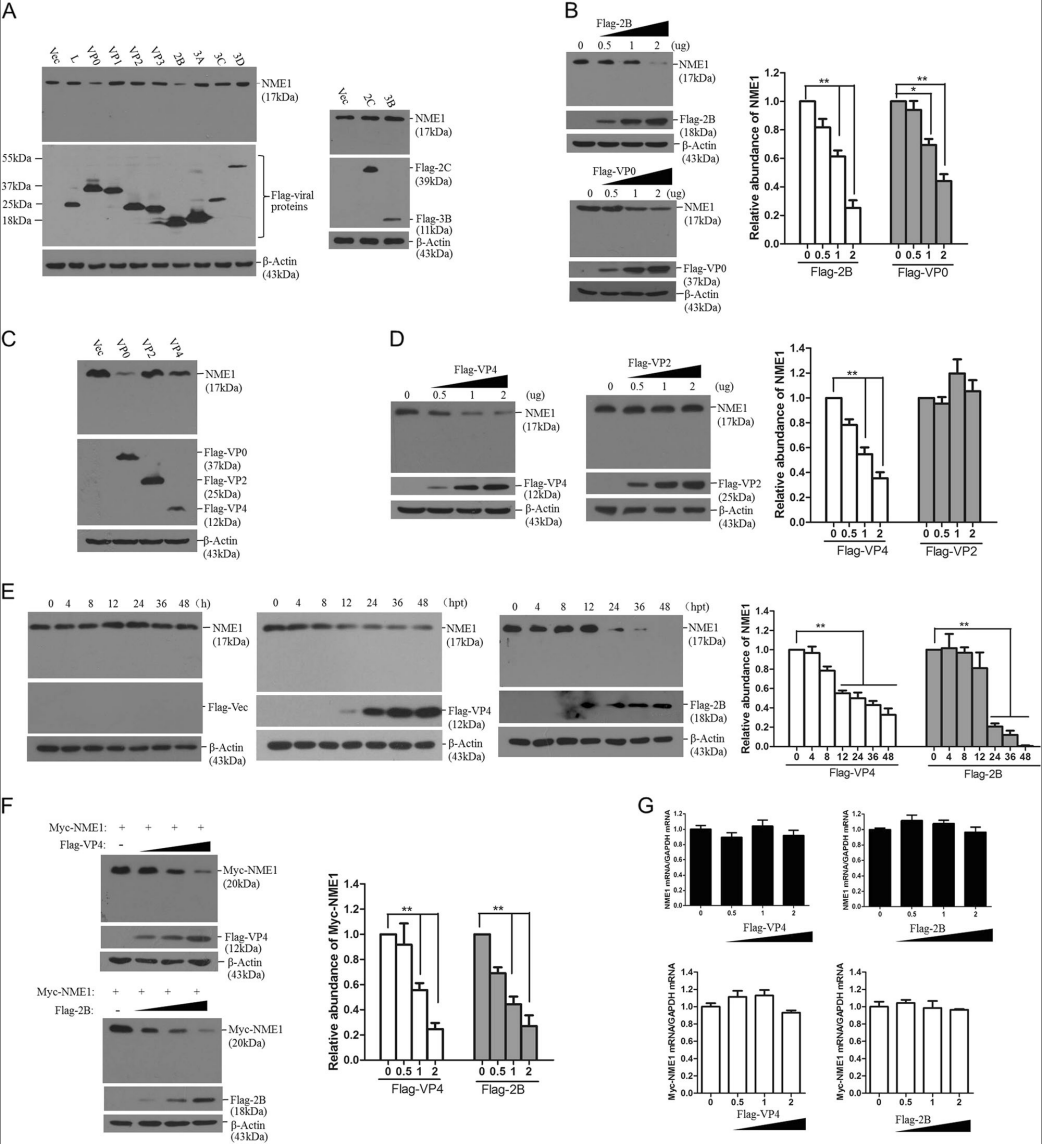
FMDV VP4 and 2B induce reduction of NME1. **a** PK-15 cells were transfected with 2 μg empty vector plasmid or 2 μg plasmids expressing various Flag-tagged viral proteins. The transfected cells were collected at 48 hpt and subjected to Western blotting. **b** PK-15 cells were transfected with increasing amounts of Flag-2B- or Flag-VP0-expressing plasmids (0, 0.5, 1, or 2 μg). Empty vector was used in the transfection process to ensure that the cells received the same amounts of total DNA plasmids. Expression of NME1 and Flag-2B or Flag-VP0 was detected by Western blotting. Relative fold-change in abundance of NME1 protein in the transfectants was determined by densitometric analysis. **c** PK-15 cells were transfected with 2 μg empty vector, Flag-VP0-, Flag-VP4-, or Flag-VP2-expressing plasmids. The transfected cells were collected at 48 hpt and subjected to Western blotting analysis. **d** PK-15 cells were transfected with increasing amounts of Flag-VP4- or Flag-VP2-expressing plasmids (0, 0.5, 1, or 2 μg). Empty vector was used in the transfection process to ensure that the cells received the same amounts of total DNA plasmids. Expression of NME1 and Flag-VP4 or Flag-VP2 were detected by Western blotting. Relative fold-change in abundance of NME1 protein in the transfectants was determined by densitometric analysis. **e** PK-15 cells were transfected with 2 μg empty vector, Flag-VP4- or Flag-2B-expressing plasmids, and the transfected cells were harvested at 0,4, 8, 12, 24, 36, and 48 hpt. Expression of NME1 and Flag-VP4 or Flag-2B was detected by Western blotting. Relative fold-change in abundance of NME1 protein in the transfectants was determined by densitometric analysis. **f** HEK-293T cells were co-transfected with 2 μg Myc-NME1-expressing plasmid and different amounts of Flag-VP4 or Flag-VP2-expressing plasmids (0, 0.5, 1, or 2 μg). Expression of Myc-NME1, Flag-VP4, or Flag-2B was detected by Western blotting using anti-Myc or anti-Flag antibodies. Relative fold-change in abundance of Myc-NME1 protein in the transfectants was determined by densitometric analysis. **g** PK-15 cells were transfected with increasing amounts of Flag-VP4 or Flag-2B-expressing plasmids (0, 0.5, 1, or 2 μg). Expression of NME1 mRNA was detected by qPCR (Upper panel). PK-15 cells were transfected with 2 μg Myc-NME1 plasmids and increasing amounts of Flag-VP4- or Flag-2B-expressing plasmids (0, 0.5, 1, or 2 μg). Expression of Myc-NME1 mRNA was detected by qPCR (Lower panel). Empty vector was used in the above transfection process to ensure that the cells received the same amounts of total DNA plasmids. All the experiments were repeated three times

FMDV infection decreased NME1 expression. To screen the viral proteins that were responsible for FMDV-induced NME1 decrease, PK-15 cells were transfected with the empty vector plasmid or plasmids expressing various Flag-tagged viral proteins. The expression of endogenous NME1 was detected by Western blotting. VP0 and 2B proteins significantly decreased NME1 expression, while the other viral proteins had no effect (Fig. 4a). In dose-response experiments, PK-15 cells were transfected with increasing amounts of VP0-expressing or 2B-expressing plasmids, and the amount of NME1 was detected by Western blotting at 48 hpt. Both VP0 and 2B decreased NME1 in a dose-dependent manner (Fig. 4b). VP0 is the precursor protein of VP4 and VP2. We constructed VP4- and VP2-expressing plasmids and found that VP4, but not VP2, decreased NME1 (Fig. 4c). These dose-dependent experiments confirmed that VP4 was responsible for the VP0-induced reduction in NME1 (Fig. 4d). A time course assay showed that VP4 and 2B decreased NME1 over time, and no cleaved bands were observed; and NME1 was not changed in the empty vector transfected cells (Fig. 4e). The suppressive role of VP4 and 2B on exogenous NME1 was subsequently evaluated. We found that both VP4 and 2B decreased Myc-NME1 in a dose-dependent manner (Fig. 4f). To investigate whether VP4- and 2B-induced decreases in NME1 were the result of decreases in mRNA expression, the amount of NME1 mRNA in Flag-VP4 or Flag-2B transfected cells was measured by qPCR. Results indicated that there was no significant decrease in NME1 mRNA levels (Fig. 4g). The effect of Flag-VP4 or Flag-2B on Myc-NME1 mRNA expression was also evaluated by co-transfection of Flag-VP4 or Flag-2B and Myc-NME1-expressing plasmids, and the qPCR analysis also suggested that Flag-VP4 or Flag-2B did not decrease NME1 mRNA (Fig. 4g). This implied that both VP4 and 2B decreased NME1 at protein levels.

### FMDV VP4 protein suppresses NME1 expression which impairs p53-mediated signaling

**Fig. 5:**
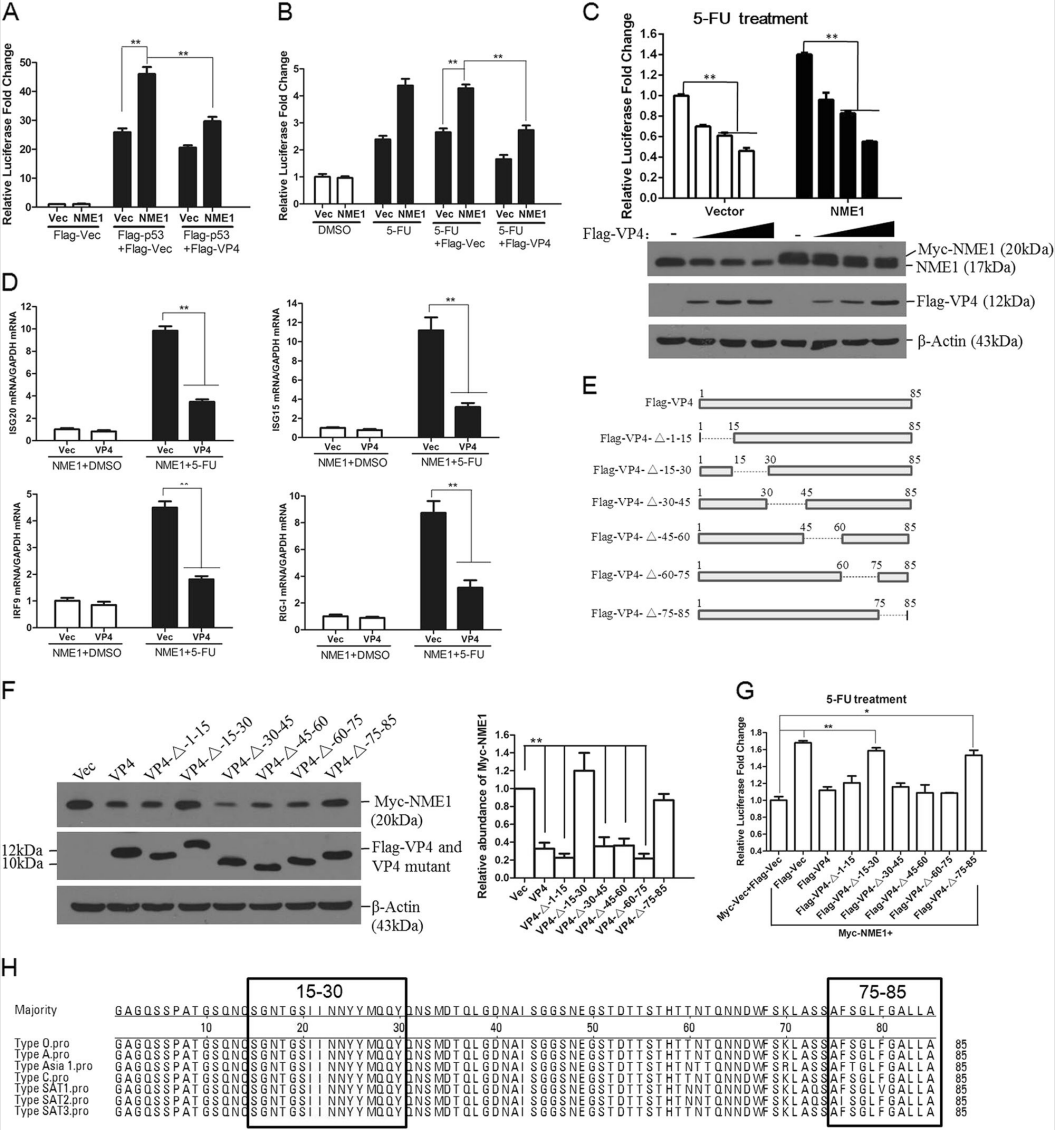
FMDV VP4 impairs NME1-enhanced p53 transcriptional activity. **a** HEK-293T cells cultured in 24-well plates were co-transfected with 0.2 μg Myc-vector or 0.2 μg Myc-NME1 and 0.1 μg Flag-vector or 0.1 μg Flag-p53 plasmids with Flag-vector (0.2 μg) or Flag-VP4 plasmids (0.2 μg), along with 0.1 μg p53 luciferase reporter plasmid. pRL-TK *Renilla* luciferase reporter plasmid (0.01 μg) was used in the reporter assay to normalize the transfection efficiency. The cells were collected at 24 hpt and luciferase activities were measured using the dual-specific luciferase assay kit. **b** HEK-293T cells cultured in 24-well plates were co-transfected with 0.2 μg Myc-vector or 0.2 μg Myc-NME1, and 0.2 μg Flag-vector or 0.2 μg Flag-VP4 plasmids, along with 0.1 μg p53 luciferase reporter plasmid and 0.01 μg pRL-TK plasmid. The transfected cells were incubated with DMSO or 5-FU (20 μg/mL) at 6 hpt, the luciferase activities were measured after 24 h incubation. **c** HEK-293T cells cultured in 24-well plates were co-transfected with 0.2 μg Myc-vector or 0.2 μg Myc-NME1, and increasing amounts of Flag-VP4 plasmids (0, 0.1, 0.2, or 0.4 μg), along with 0.1 μg p53 luciferase reporter and 0.01 μg pRL-TK plasmids. The transfected cells were incubated with 5-FU at 6 hpt, the luciferase activities were measured after 24 h incubation. Expression of Myc-NME1 and NME1 were detected by Western blotting using anti-NME1 antibody. Expression of Flag-VP4 was detected using anti-Flag antibody. **d** HEK-293T cells cultured in six-well plates were co-transfected with 2 μg Flag-vector or 2 μg Flag-VP4, and 2 μg Myc-NME1 plasimds. The transfected cells were treated with DMSO or 5-FU at 6 hpt for another 24 h. mRNA expression levels of *ISG20*, *IRF9*, *RIG-I,* and ISG15 were detected by qPCR. **e** Schematics of a series of Flag-tagged truncated VP4 constructs. **f** HEK-293T cells were co-transfected with Myc-NME1 and Flag-vector, Flag-tagged VP4 or VP4 mutant plasmids. Expression of My-NME1 was detected by Western blotting using anti-Myc antibody. Expression of Flag-VP4 and VP4 mutants was detected using anti-Flag antibody. Relative fold-change in abundance of Myc-NME1 protein in the transfectants was determined by densitometric analysis. **g** HEK-293T cells cultured in 24-well plates were co-transfected with 0.2 μg Myc-vector or 0.2 μg Myc-NME1, and Flag-VP4 or Flag-tagged VP4 mutants plasmids (0.2 μg), along with 0.1 μg p53 luciferase reporter and 0.01 μg pRL-TK plasmids. The transfected cells were incubated with 5-FU at 6 hpt, and luciferase activities were measured after 24 h incubation. All the experiments were repeated at least three times. **h** Amino acid alignment of different serotypes of FMDV VP4 coding sequences using LaserGene software (http://www.dnastar.com/). The GenBank accession numbers of the viral VP4 sequences were: Type O (AET43040.1), Type A (ADR66173.1), Type Asia 1 (ABF74751.2), Type C (ARO74648.1), Type SAT1 (ARO74655.1), Type SAT2 (ARO74650.1), and Type SAT3 (ALJ79273.1)

FMDV 2B inhibits the expression or functions of several host proteins^21,22^. Perhaps for the first time, we observed a FMDV VP4-induced reduction in host proteins. Therefore, further studies were performed to investigate the novel antagonistic role of VP4. NME1-enhanced p53-mediated transcriptional activity. VP4 decreased NME1 expression. To investigate the effect of VP4 on p53-mediated transcriptional activity, HEK-293T cells were co-transfected with Myc-vector or Myc-NME1, and Flag-vector or Flag-p53, as well as Flag-VP4-expressing plasmids in the presence of the p53-Luc reporter and the pRL-TK plasmids. The transfectants were harvested at 24 hpt and subjected to luciferase assays. NME1-enhanced p53-Luc promoter activity was markedly impaired by VP4 (Fig. 5a). The suppressive effect of VP4 on 5-FU-induced p53-Luc promoter activation was also evaluated, which suggested that VP4 blocked NME1-enhanced p53-Luc promoter activity (Fig. 5b). The dose-response experiment was further performed. NME1 promoted 5-FU-triggered p53-Luc promoter activation. However, VP4 impaired this activation in a dose-dependent manner (Fig. 5c).

The regulatory effect of VP4 on 5-FU-induced upregualtion of *ISG20*, *IRF9*, *RIG-I*, and *ISG15* in the presence of NME1 was subsequently examined, and results showed that VP4 significantly suppressed expression of these IFN-inducible genes (Fig. 5d). A series of VP4-truncated mutants were constructed to investigate the functional region of VP4 that was responsible for reduced NME1 expression (Fig. 5e). Unexpectedly, deletion of the 15–30 region of VP4 significantly slowed gel electrophoresis of VP4. The 15–30 and 75–85 regions of VP4 were found to be crucial for reduction of NME1 (Fig. 5f). The regulatory role of VP4 mutants in NME1-enhanced p53 signaling was also analyzed. HEK-293T cells were co-transfected with Myc-Vector or Myc-NME1-expressing plasmids and Flag-Vector, and Flag-VP4 or Flag-VP4 mutants expressing plasmids in the presence of p53-Luc reporter and pRL-TK plasmids. Transfected cells were incubated with 5-FU for 24 h at 6 hpt, and were collected and subjected to luciferase assays. VP4 significantly inhibited NME1 expression and resulted in decreased p53 signaling transduction. However, the deletion of the 15–30 or 75–85 regions of VP4 restored NME1-mediated enhancement of p53 signaling (Fig. 5g). The VP4 amino acids of seven serotypes of FMDV were aligned, which suggested that VP4 proteins were highly conserved among the different FMDV serotypes, and the 15–30 and 75–85 regions were also very conservative (Fig. 5h). These results confirmed that FMDV VP4 suppressed NME1 expression, thus inhibiting p53-mediated signaling. Deletion of the crucial regions that were responsible for causing the reduction of NME1 inhibited the antagonistic effect of VP4 on p53 signaling.

### VP4 decreases NME1 and promotes interaction of p53 with MDM2

**Fig. 6:**
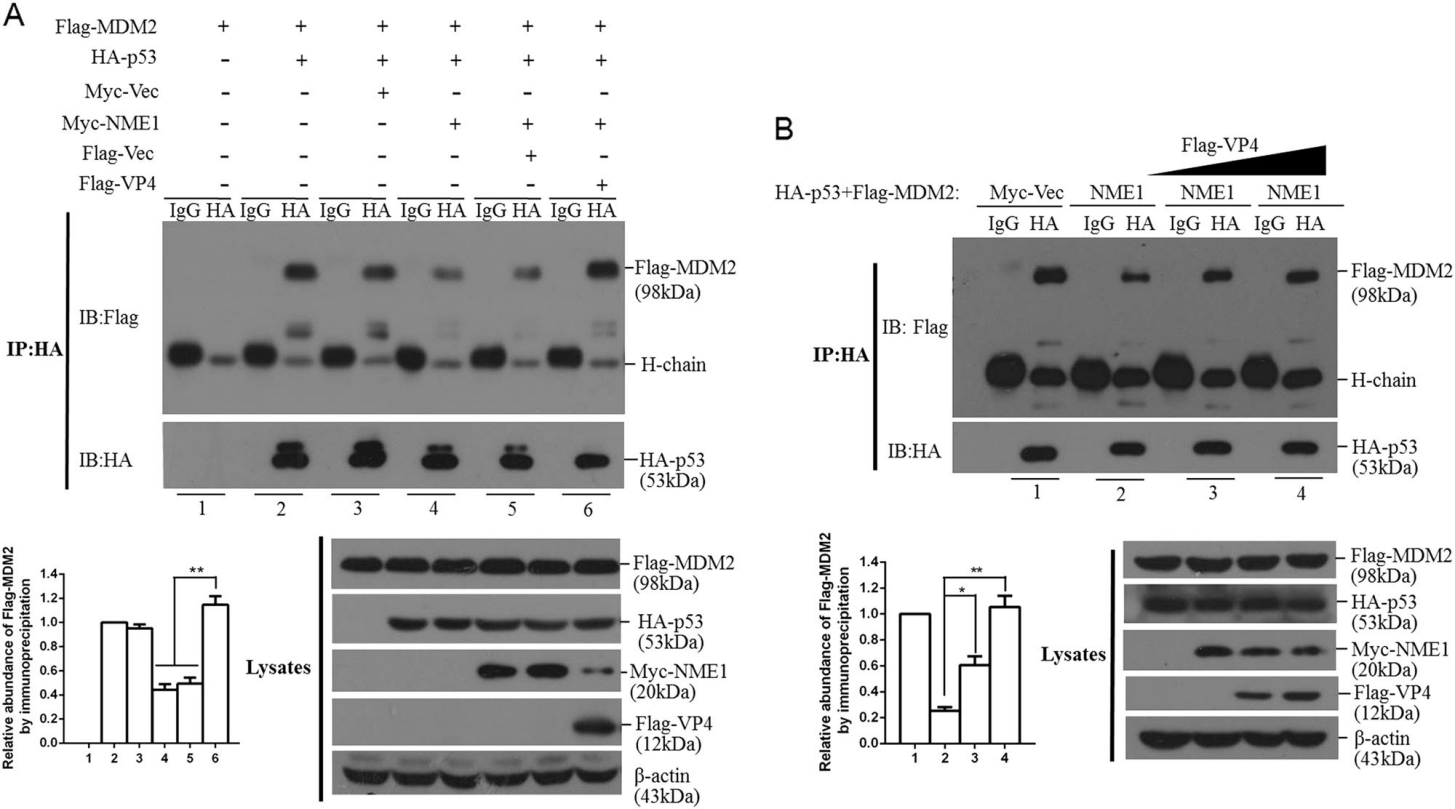
FMDV VP4 induces NME1 reduction to promote interaction of p53 with MDM2. **a** HEK-293T cells grown in 10-cm dishes were co-transfected with 6 μg Flag-MDM2 plasmid with the indicated plasmids (6 μg), and MG132 was added at 20 μM. The cells were collected and lysed at 24 hpt and immunoprecipitated with HA antibody. Expression of Flag-MDM2 and HA-p53 was detected using appropriate antibodies. Relative fold-change in abundance of Flag-MDM2 was determined by densitometric analysis. Whole cell lysates were also analyzed by Western blotting using appropriate antibodies. **b** HEK-293T cells grown in 10-cm dishes were co-transfected 6 μg Flag-MDM2 plasmid, 6 μg HA-p53 plasmid, 6 μg Myc-vector or Myc-NME1 plasmids, and 6 μg Flag-vector or different doses of Flag-VP4 plasmids (0, 3, or 6 μg). MG132 was added at 20 μM. The cells were collected and lysed at 24 hpt and immunoprecipitated with HA antibody. Expression of Flag-MDM2 and HA-p53 was detected using appropriate antibodies. Relative fold-change in abundance of Flag-MDM2 was determined by densitometric analysis. Whole cell lysates were also analyzed by Western blotting using appropriate antibodies. All the experiments were repeated three times

NME1 plays a significant role in regulation of p53 function^23,24^. NME1 stabilizes p53 by interacting with p53 and suppressing the interaction of p53 with MDM2^25^. To investigate VP4-induced negative regulation of p53 activity, we evaluated interaction of p53 with MDM2 in the presence or absence of Myc-NME1 and Flag-VP4. HEK-293T cells were co-transfected with Flag-MDM2, HA-p53, Myc-Vector or Myc-NME1, and Flag-Vector or Flag-VP4 plasmids in the presence of MG132. The cells were collected and lysed at 24 hpt and immunoprecipitated with hemagglutinin (HA) antibody. NME1 significantly blocked p53-MDM2 interaction, however, VP4 alleviated the NME1-mediated blocking effect (Fig. 6a). VP4 impaired NME1-mediated inhibition of the p53-MDM2 interaction in a dose-dependent manner (Fig. 6b). This suggested that VP4 antagonized p53 signaling by decreasing NME1, which destabilized p53 and attenuated p53 signaling.

### FMDV VP4 induces NME1 reduction by the lysosomal pathway

**Fig. 7:**
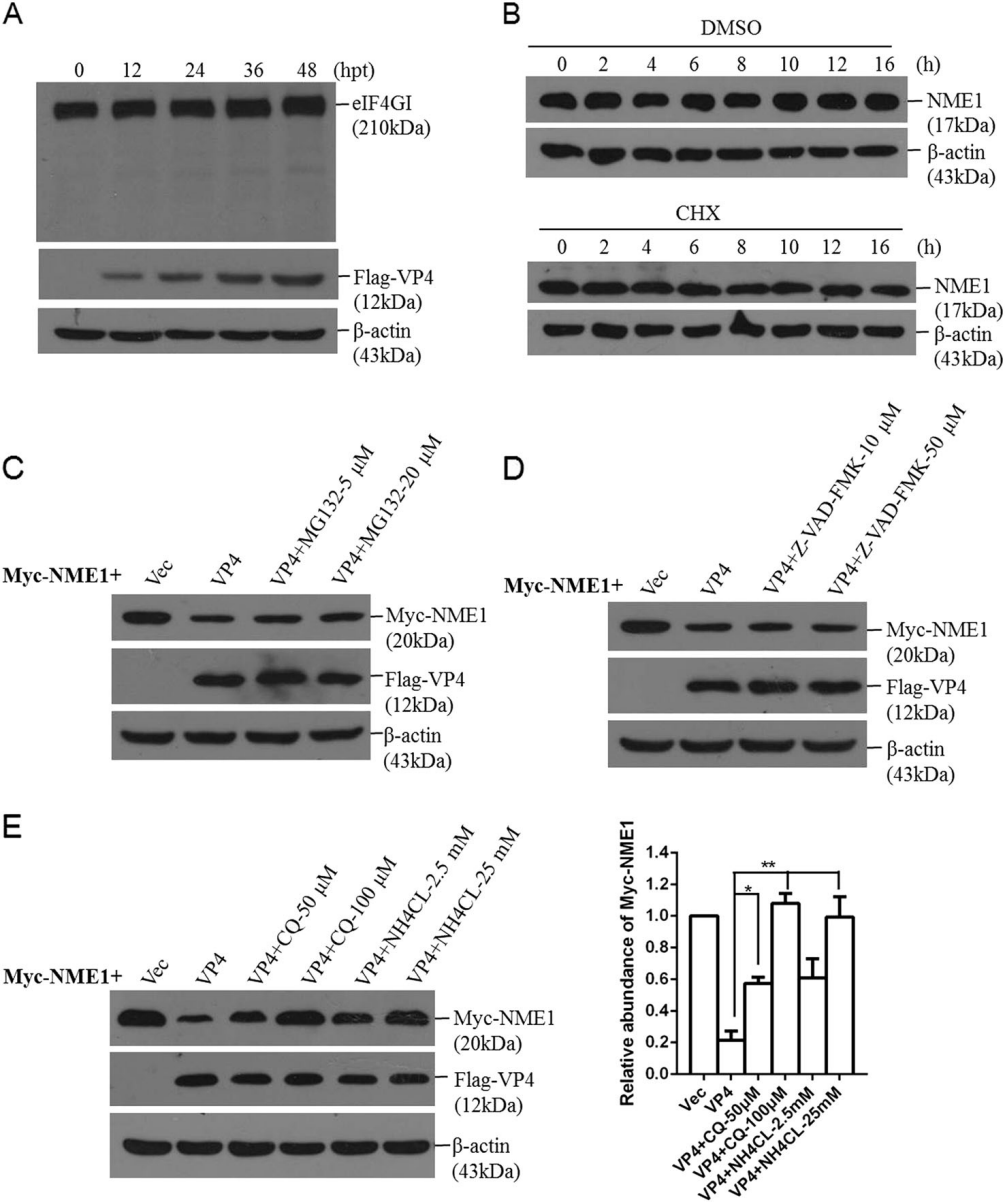
FMDV VP4 induces NME1 reduction through lysosomal pathway. **a** PK-15 cells were transfected with 2 μg Flag-VP4 expressing plasmid. The cells were collected at 0, 12, 24, 36, or 48 hpt. Expression of cellular eIF4G was detected using anti-eIF4G antibody. Expression of Flag-VP4 was detected using anti-Flag antibody. **b** PK-15 cells were treated with with DMSO or CHX (50 μg/mL) for 0, 2, 4, 6, 8, 10, 12, and 16 h. Expression of NME1 was detected by Western blotting using anti-NME1 antibody. **c**-**e** HEK-293T cells grown in 6-well plates were co-transfected with 2 μg Myc-NME1, 2 μg Flag-vector, or Flag-VP4 plasmids in the presence or absence of (c) MG132 (5 or 20 μM), (**d**) Z-VAD-FMK (10 or 50 μM), (**e**) CQ (50 or 100 μM) or NH_4_CL (2.5 or 25 mM). The cells were collected at 24 hpt. Expression of Myc-NME1 was detected by Western blotting using anti-Myc antibody. Expression of Flag-VP4 was detected using anti-Flag antibody. Relative fold-change in abundance of Myc-NME1 protein was determined by densitometric analysis. All the experiments were repeated at least three times

FMDV infection leads to the cleavage of host eukaryotic translation initiation factor 4G (eIF4G) to shut off synthesis of several host proteins. Hence, we investigated whether expression of VP4 resulted in the cleavage of eIF4G. The integrity of eIF4G in VP4-overexpressing cells was evaluated. We found that VP4 did not cause the cleavage of eIF4G (Fig. 7a). The translation inhibitor cycloheximide (CHX) was used to confirm this correlation. PK-15 cells were treated with DMSO or CHX for 0, 2, 4, 6, 8, 10, 12, and 16 h, and NME1 levels were detected by Western blotting. Inhibition of host protein synthesis did not result in significant reduction in NME1 at 16 h (Fig. 7b). These results suggest that FMDV-induced or VP4-induced reduction of NME1 protein was not related to the well-characterized blocking effect of FMDV on cellular translation (viral host cell shutoff). The inhibitors of the proteasome, lysosome, and caspase pathways were further used to investigate the pathway that was involved in the VP4-induced decrease of NME1. HEK-293T cells were co-transfected with Myc-NME1 and Flag-Vector or Flag-VP4-expressing plasmids in the absence or presence of MG132, Z-VAD-FMK, CQ or NH_4_CL for 24 h. Expression of Myc-NME1 was detected by Western blotting (Fig. 7c–e). Incubation with CQ and NH_4_CL restored levels of Myc-NME1, which indicated that the VP4-induced decrease of NME1 was dependent on the lysosome pathway (Fig. 7e).

### VP4 does not interact with NME1 but degrades NME1 through macroautophagy pathway to promote FMDV replication

**Fig. 8:**
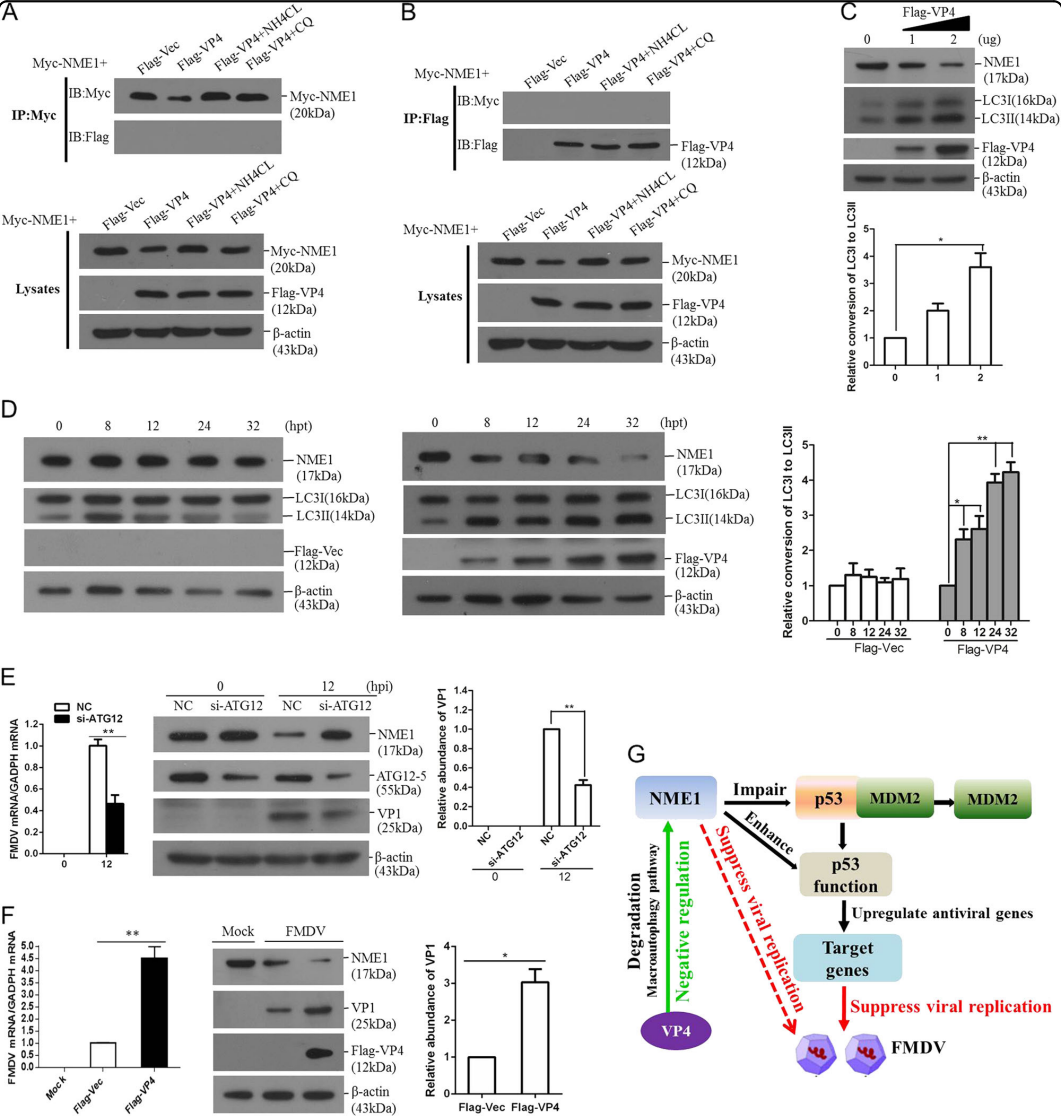
FMDV VP4 does not interact with NME1. **a** HEK-293T cells grown in 10-cm dishes were co-transfected 6 μg Myc-NME1 plasmids, 6 μg Flag-vector or Flag-VP4 plasmids in the presence of NH_4_CL (25 mM) or CQ (100 μM). The cells were collected and lysed at 36 hpt and were immunoprecipitated with Myc antibody. Expression of Myc-NME1 and Flag-VP4 was detected using appropriate antibodies. Whole cell lysates were also analyzed by Western blotting using appropriate antibodies. **b** Reversed immunoprecipitation was performed using Flag antibody. All the proteins were detected using appropriate antibodies. **c** PK-15 cells were transfected with 0, 1, or 2 μg of Flag-VP4-expressing plasmid. The empty vector was used in the transfection process to ensure that the same amount of plasmid was transfected into the same number of cells. The expression of NME1 and Flag-VP4 was immunoblotted. Autophagic flux was detected by LC3II turnover using Western blotting analysis. Relative fold-change in conversion of LC3I to LC3II protein was determined by densitometric analysis. **d** PK-15 cells were transfected with the empty vector or Flag-VP4 plasmids for 0, 8, 12, 24, or 32 h. The expression of NME1 and the conversion of LC3I to LC3II was measured by Western blotting analysis. Relative fold-change in conversion of LC3I to LC3II protein was determined by densitometric analysis. **e** PK-15 cells were transfected with negative control (NC) siRNA or siRNA targeting ATG12 (si-ATG12) for 24 h, and the cells were infected with equal amounts of FMDV for 12 h. The expression of FMDV RNA was detected by qPCR. The expression of ATG12-5, NME1, and FMDV VP1 proteins was detected by Western blotting. Relative fold-change in VP1 protein was determined by densitometric analysis. **f** PK-15 cells were transfected with 2 μg of empty vector or Flag-VP4-expressing plasmids for 24 h. The cells were infected with equal amounts of FMDV for 12 h. The viral RNA were analyzed by qPCR. NME1 and FMDV VP1 proteins were detected by Western blotting. Relative fold-change in VP1 protein was determined by densitometric analysis. All the above experiments were repeated at least three times. **g** Model of FMDV VP4-mediated indirect antagonistic effect on p53-induced antiviral response by lysosomal degradation of host NME1 protein

We investigated whether there was a direct interaction between VP4 and NME1. HEK-293T cells were co-transfected with the Myc-NME1 plasmid and Flag-vector or Flag-VP4, and the lysosomal inhibitor CQ or NH_4_CL was used to alleviate VP4-induced reduction of NME1. NME1 did not immunoprecipitate VP4, regardless of treatment with or without lysosomal inhibitor (Fig. 8a). Reverse immunoprecipitation using anti-Flag antibody also suggested that VP4 did not immunoprecipitate NME1 (Fig. 8b). This indicated that VP4 did not interact with NME1, and VP4 might induce NME1 reduction by an indirect manner.

VP4-induced decrease of NME1 was dependent on the lysosome pathway. In order to provide more insights into this mechanism, we evaluated the effect of VP4 on macroautophagy. PK-15 cells were transfected with vector plasmids or increasing amounts of Flag-VP4 plasmids for 24 h, the expression of NME1 and the conversion of LC3I to the lipidation form LC3II was measured. The level of lipidation form LC3II was significantly increased in VP4-overexpressing cells showing a dose-dependent manner. In contrast, the NME1 expression was gradually decreased (Fig. 8c). The time course experiment was also performed. The decrease of NME1 and conversion of LC3I to LC3II were only observed in the VP4-overexpressing cells, but was not changed in the vector plasmids transfected cells (Fig. 8d). This indicated that VP4 might induce the macroautophagy and resulted in NME1 degradation. To further investigate the role of macroautophagy on NME1 degradation, the critical protein of autophagy ATG-12 was knocked down by RNA interference (RNAi) method. The expression of NME1 in the ATG-12 knockdown cells was evaluated. Silence of ATG-12 significantly restored NME1 expression during FMDV infection, and the restored expression of NME1 resulted in decreased FMDV replication (Fig. 8e). This suggests that the macroautophagy pathway is essential for NME1 degradation during FMDV infection.

The viral replication status and NME1 protein levels in VP4-overexpressing cells were further investigated. Both the viral RNA and protein levels were significantly higher in VP4-overexpressing cells than that in the vector-transfected cells. In contrast, a considerable decreased amounts of NME1 was observed in VP4-overexpressing cells compared with that in the vector-transfected cells (Fig. 8f). This confirmed that VP4 degraded NME1 and promoted FMDV replication.

## Discussion

NME1 is a well-characterized metastasis suppressor. Low levels of NME1 expression have been linked to advanced stages of ovarian carcinoma and lymph node metastasis^26^. Transfer of the NME1 gene to inhibit metastasis is a promising therapeutic strategy^27^. NME1 interacts with p53 and activates p53 function during anti-tumor progression^28^, while p53 plays critical roles in cell cycle arrest, differentiation, and apoptosis, and contributes to tumor suppression^29^. It also regulates expression of many antiviral genes and performs antiviral functions^14,30^. NME1 regulates p53 function in tumor cells and has an anti-tumor function. Whether NME1 is involved in the host antiviral response remains unknown. We demonstrated that NME1-enhanced p53-mediated transcription. The antiviral-related genes including *ISG20*, I*RF9*, *RIG-I*, and *ISG15* were upregulated by overexpression of NME1. ISG20 and ISG15 possess direct antiviral activity, and have been shown to mediate protection in several different viral infection models^31–33^. RIG-I and IRF9 are involved in type I IFN production and perform important regulatory functions in host antiviral responses^34,35^. This indicates that NME1 regulates the host antiviral response and possesses an antiviral function. This is believed to be the first demonstration of the antiviral-activity of NME1.

NME1 interacts with MIF and significantly alleviates the MIF-mediated repressive effect on p53 activity. NME1 also interacts with the central DNA-binding domain of p53 to remove MDM2, a negative regulator of p53, from the p53-MDM2 complex, which is involved in the p53-induced antiviral response^6^. This indicates that NME1 plays a significant regulatory role in p53-mediated functions. Silencing of NME1 results in activation of the mitogen activated protein kinase (MAPK) signaling pathway. Many viruses manipulate MAPK pathways during viral replication, and activation of the MAPK pathway is required for viral replication^36–38^. NME1 might also affect viral replication through regulation of MAPK pathway activation. In this study, NME1 enhanced p53-mediated transcription and increased antiviral gene expression. Therefore, FMDV reduces NME1 in the infected cells to impair host antiviral effect. The two viral proteins 2B and VP4 contributed to the reduction of NME1. FMDV 2B protein exerts a suppressive role on host protein expression^22^. However, for the first time, the VP4-induced degradation of host protein by the macroautophagy pathway was determined. Previous study reports that FMDV utilizes the macroautophagy pathway during viral replication^39^. We determined that VP4 degraded NME1 by macroautophagy pathway and resulted in FMDV replication. This confirmed the positive regulative role of the autophagic pathway for FMDV replication.

No interaction was detected between VP4 and NME1, suggesting that VP4 did not directly interrupt the interaction of NME1 with MIF or p53. VP4 protein suppresses NME1 expression through a lysosomal pathway and promotes interaction of p53 with MDM2. This indicates that VP4 antagonizes p53-mediated host antiviral effect by targeting NME1 protein. The decrease of NME1 expression subsequently impairs p53-mediated transcriptional activation and increases FMDV replication. Activation of the MAPK signaling pathway promotes enterovirus 71 infection in immature dendritic cells^40^. Downregulation of NME1 might also activate the MAPK pathway and promote virus replication. Besides, the low expression levels of NME1 or p53 are associated with tumorigenesis^41^. Low expression of NME1 also results in decreased antiviral activity in host cells, which indicates why patients with tumors are more susceptible to viral infection. Besides, we also investigated if the catalytic activity of the NME1 kinase is important for its anti-viral activity during FMDV infection by construction of different NME1 mutants. P96S and S120G mutation exhibited reduced histidine kinase activity. The E129A completely lost NDPK activity. H118F is a kinase-dead mutation that impairs both diphosphate kinase (NDPK) and histidine kinase activities^18,19^. Mutation of H118 impaired the antiviral role of NME1, suggesting an important role of this site. H118 plays significant role in suppression of the neoplastic transformation and tumorigenesis by NME1^19^. This indicates that H118 plays very important roles in the host cells. Mutation of P96, S120, or E129 did not affect the antiviral role of NME1, which suggested that abrogation of the NDPK activity or histidine kinase activity separately did not affect antiviral role of NME1. However, mutation of the NDPK activity or histidine kinase activity simultaneously impaired the antiviral role of NME1. All these data suggest a complicated antiviral mechanism of NME1.

Viral proteins often perform antagonistic roles against host antiviral responses to facilitate viral replication^42,43^. The p53-mediated antiviral pathway is targeted by various viruses. The NS1 protein of influenza A virus binds to p53 and suppresses the transcriptional activity of p53^44^. Hepatitis B virus X protein binds with p53, inhibiting its sequence-specific DNA binding, and inhibits p53-mediated transcriptional activation^45^. In this study, we identified a new strategy for viruses to antagonize p53-mediated function. FMDV VP4 induces lysosomal degradation of NME1 resulting in decreased p53 transcriptional function (Fig. 8g). This suggests an indirect antagonistic effect induced by FMDV on the p53 pathway.

FMDV structural proteins VP1 and VP3, and non-structural proteins L^pro^, 2B, 2C, 3A, and 3C^pro^, interact with host proteins and promote viral replication^46–51^. We showed that FMDV VP4 is a new antagonistic factor for FMDV to suppress host antiviral response and promote viral replication. In conclusion, we showed that the tumor suppressor protein NME1 possesses antiviral activity by regulation of the p53-mediated innate inmmue antiviral response. FMDV VP4 induces degradation of NME1 using the macroautophagy pathway and counteracts the NME1-mediated antiviral effect.

## Materials and methods

### Viruses and cells

The FMDV strains O/BY/CHA/2010 (serotype O) and A/HuBWH/CHA/2009 (serotype A) were used for viral challenge experiments. The two strains were obtained from the National Foot and Mouth Diseases Reference Laboratory, Lanzhou Veterinary Research Institute, Chinese Academy of Agricultural Sciences. PK-15 cells, BHK-21, and HEK-293T cells were grown as monolayers in Dulbecco’s modified Eagle’s medium (DMEM, Invitrogen), supplemented with 10% fetal bovine serum (FBS) and then cultured at 37 °C under 5% CO_2_. The viruses used for viral challenge were propagated in BHK cells.

### Plasmids and antibodies

The full-length porcine NME1 cDNA was amplified and inserted into pcDNA^TM^3.1/myc-His(-)A vector (Invitrogen) to generate a plasmid expressing Myc-tagged NME1 (Myc-NME1). A series of plasmids expressing Flag-tagged viral proteins were constructed as described previously^52^. HA-tagged p53 plasmid (HA-p53) was constructed by inserting full-length p53 cDNA into pCAGGs vector (including a C-terminal HA tag). Flag-p53, p53-Luc reporter plasmids and control plasmid Renilla luciferase pRL-TK were kindly provided by Zhiyong Ma (Shanghai Veterinary Research Institute, Chinese Academy of Agricultural Sciences, China)^53^. All the expressing plasmids used in this study were analyzed and verified by DNA sequencing.

Commercial antibodies included: mouse monoclonal anti-Myc, monoclonal anti-Flag, rabbit polyclonal anti-NME1, and mouse monoclonal anti-β-actin (Santa Cruz Biotechnology); mouse monoclonal anti-hemagglutinin (HA) (BioLegend); mouse monoclonal anti-Flag (Sigma); rabbit polyclonal anti-eukaryotic translation initiation factor 4 gamma (eIF4G) (Abcam); rabbit polyclonal anti-LC3B (Sigma); rabbit monoclonal anti-ATG12 (Cell signaling technology); rabbit polyclonal anti-VP1 antibody was prepared by our laboratory as described previously^48^.

### Viral infection and TCID_50_ assay

Viral infection was performed as described previously^22^. Briefly, the monolayer cells were washed with PBS for three times and then incubated with FMDV at an MOI of 1. The supernatant was removed and replaced with fresh medium supplemented with 1% FBS after 1 h incubation with the viruses. For mock infection, the cells were incubated with serum-free medium instead of the virus, and the experiment was performed similarly as the viral infection method. The samples were collected at different times post-infection as required. The viral titers were determined by TCID_50_ assay as described previously^48^. The TCID_50_ values were calculated by the Reed-Muench method.

### Coimmunoprecipitation and western blotting

HEK-293T cells were grown in 10-cm dishes, and monolayer cells were transiently co-transfected with various indicated plasmids. The transfectants were harvested at 24 or 36 post-transfection (hpt) and lysed using lysis buffer as described previously^54^. The lysates were immunoprecipitated with anti-Myc or anti-Flag, and the precipitates were analyzed using Western blotting. The lysed protein samples were resolved by SDS-PAGE and transferred onto a nitrocellulose membrane (Pall). The target proteins were detected by the appropriate antibodies and visualized by enhanced chemiluminescence reagent (Pierce).

### RNA extraction and qPCR analysis

Total RNA was extracted from the cells using TRIzol reagent (Invitrogen) according to the instruction of the manufacture. The extracted RNAs were used as templates to direct the synthesis of the first-strand cDNA. M-MLV reverse transcriptase (Invitrogen) and random hexamer primers (TaKaRa) were used for PCR. The relative abundance of the synthesized cDNA was used as an indicator of target transcripts. qPCR was carried out using SYBR Premix Ex Taq (Takara). The glyceraldehyde-3-phosphate dehydrogenase (GAPDH) was used for normalization of total input RNA. Relative transcript levels were calculated using 2^−ΔΔCT^ method as described previously^54^. All the qPCR experiments were performed in triplicate and repeated three times.

### Knockdown of NME1 using siRNA

The RNAi method was used to downregulate NME1 expression in cells. The small interfering RNAs (siRNAs) used in this study were synthesized and purchased from Genepharma Company (China). The synthesized sequence for porcine NME1 was Forward: 5′-GCACCUUCAUUGCCAUCAATT-3′, Reverse: 5′-UUGAUGGCAAUGAAGGUGCTT-3′. The non-targeting siRNA (NC siRNA) was used as a negative control for NME1 siRNA. The transfection method was carried out as described previously^22^.

### Reporter gene assays

HEK-293T cells were grown in 24-well plates. p53-Luc reporter plasmid and control plasmid Renilla luciferase pRL-TK were transfected using Lipofectamine 2000 (Invitrogen) as previously described^48,53^. The empty vector was used in the transfection process to ensure that the same amount of plasmid was transfected into the same number of cells. The control plasmid Renilla luciferase pRL-TK was used in the reporter gene assay to normalize transfection efficiency. To analyze the effect of NME1 and viral proteins on p53 transcriptional activity, HEK-293T cells were transfected with the indicated expression plasmids in the presence of the p53-Luc reporter and pRL-TK plasmids. The collected cells were lysed using the passive lysis buffer (Promega) and subjected to measurement of luciferase activity using the Dual-Luciferase Reporter Assay Systems (Promega). 5-FU was purchased from Sigma and dissolved in DMSO, the dissolved 5-FU was added at 20 μg/mL to the medium and incubated for the indicated times.

### Chemical inhibitors assay

CHX (dissolved in DMSO), a protein synthesis inhibitor, was purchased from Cell Signaling Technology and used to investigate the eIF4G-independent suppressive effect induced by FMDV proteins. CHX (50 μg/mL) was added to the culture medium, and the cells were harvested at the indicated time points and subjected to Western blotting. The proteasome inhibitor MG132 (Merck; dissolved in DMSO), the lysosomal inhibitor CQ (Sigma; dissolved in fresh culture medium) or NH_4_CL (Sigma; dissolved in water), and the caspase inhibitor Z-VAD-FMK (Sigma; dissolved in DMSO) were used to determine the pathways that were responsible for decreased host protein expression. The cells were maintained in the culture medium in the presence or absence of MG132 (5 or 20 μM), CQ (50 or 100 μM), NH_4_CL (2.5 or 25 mM) or Z-VAD-FMK (10 or 50 μM). The collected cells were subjected to Western blotting.

### Densitometric analysis

Densitometric analyses of Western blotting strips were performed using Quantity One software (Bio- Rad) and normalized to β-actin. As for the conversion of LC3I to the lipidation form LC3II. The ratio LC3II/LC3I was calculated and then normalized to β-actin. The relative fold changes are presented as the means ± S.E. from three independent experiments.

### Statistical analysis

All the measured results in this study were expressed as the mean values ± standard error (mean ± SE) of three independent experiments. The Student’s *t* test was used to analyze the significance (two-tail Student’s *t* test). *P < 0.05 was taken as statistically significant, **P < 0.01 was taken as highly significant.
